# Prevalence and Determinants of Uncontrolled Hypertension Among Treated Adults in a Rural Primary Health Care Facility in South Africa: A Cross-Sectional Study

**DOI:** 10.3390/epidemiologia7030082

**Published:** 2026-06-10

**Authors:** Guillermo Alfredo Pulido Estrada, Mercedes Nico-Garcia, Olufunmilayo Olukemi Akapo, Mirabel Kah-Keh Nanjoh

**Affiliations:** 1 School of Public Health, Faculty of Medicine and Health Sciences, Walter Sisulu University, Mthatha 5100, South Africa; gpulidoestrada@wsu.ac.za (G.A.P.E.); mnanjoh@wsu.ac.za (M.K.-K.N.); 2 Department of Family Medicine and Rural Health, Faculty of Medicine and Health Sciences, Walter Sisulu University, Mthatha 5100, South Africa; mnico-garcia@wsu.ac.za; 3 School of Pathology, Faculty of Medicine and Health Sciences, Walter Sisulu University, Mthatha 5100, South Africa

**Keywords:** hypertension control, uncontrolled hypertension, medication adherence, primary health care, South Africa

## Abstract

Background: Hypertension remains one of the leading modifiable risk factors for cardiovascular diseases globally, yet blood pressure control remains suboptimal in many low- and middle-income countries. Understanding the prevalence of uncontrolled hypertension and its associated factors is important for improving treatment outcomes, particularly in rural primary health care settings. Methods: A cross-sectional study was conducted among 103 hypertensive patients receiving follow-up care at a rural community health centre in the Eastern Cape Province of South Africa between August and October 2024. Sociodemographic, lifestyle, and clinical information were collected using a semi-structured questionnaire and medical record review. Medication adherence was assessed using the Hill–Bone Compliance to High Blood Pressure Therapy Scale. Uncontrolled hypertension was defined as systolic blood pressure ≥ 140 mmHg and/or diastolic blood pressure ≥ 90 mmHg. Associations between explanatory variables and uncontrolled hypertension were analysed using chi-square tests and multivariable logistic regression. Results: The mean age of participants was 63 ± 13 years, and 65% were female. The prevalence of uncontrolled hypertension was 63.1% (65/103), while 36.9% (38/103) achieved blood pressure control. The median systolic blood pressure was 149 mmHg (IQR: 135–163) and the median diastolic blood pressure was 84 mmHg (IQR: 76–89). Low medication adherence was significantly associated with uncontrolled hypertension (OR = 4.20, 95% CI: 1.75–10.09, *p* = 0.001). Forgetfulness and non-use of reminders were common barriers to adherence. Conclusions: Uncontrolled hypertension remains highly prevalent among treated patients in this rural setting. Low medication adherence was significantly associated with uncontrolled hypertension, suggesting that adherence support strategies warrant further investigation in similar resource-limited primary health care settings.

## 1. Introduction

Hypertension is one of the most prevalent non-communicable diseases worldwide and is defined as a systolic blood pressure of ≥140 mmHg and/or a diastolic blood pressure of ≥90 mmHg. It is estimated that approximately 1.3 billion people were living with hypertension globally in 2021, and the burden continues to rise, particularly in low- and middle-income countries [1,2,3,4]. Uncontrolled hypertension is a major modifiable risk factor for cardiovascular diseases, including stroke, coronary heart disease, myocardial infarction, and heart failure. Consequently, effective management of hypertension is critical for reducing cardiovascular-related morbidity and mortality [5,6,7,8,9,10,11,12].

Globally, improvements in hypertension treatment and management have contributed to reductions in the incidence of stroke and ischaemic heart disease. Despite these advances, blood pressure control remains suboptimal in many settings [13,14]. Recent global estimates indicate that only about one in five patients receiving treatment for hypertension achieve adequate blood pressure control. Several factors contribute to poor blood pressure control, including unhealthy lifestyle practices such as excessive salt intake, alcohol consumption, physical inactivity, and obesity, as well as socioeconomic and health system challenges [15,16].

In South Africa, hypertension represents a significant public health concern. Population-based studies have reported high prevalence rates and low levels of awareness and control among adults [17,18]. Poor hypertension control contributes substantially to the burden of cardiovascular diseases and remains one of the leading causes of mortality in the country. Rural communities may face additional challenges related to socioeconomic disadvantage, limited health resources, and barriers to long-term adherence to treatment [19,20].

Medication adherence plays a crucial role in achieving effective blood pressure control. Patients who consistently adhere to antihypertensive therapy are more likely to maintain optimal blood pressure levels and reduce their risk of cardiovascular complications [21,22]. Adherence to long-term medication regimens can be difficult for many patients due to factors such as forgetfulness, complex treatment schedules, limited health literacy, and inadequate patient support systems [23]. Emerging digital health tools and reminder systems are increasingly being explored as practical strategies to improve medication adherence and hypertension control in resource-limited settings [24,25].

Considering the disadvantaged socioeconomic conditions and limited healthcare resources often experienced by patients in rural areas of the Eastern Cape Province, it is important to better understand the factors influencing blood pressure control within these communities. Continuous monitoring of hypertension control rates and identification of associated determinants are essential for informing targeted interventions aimed at improving treatment outcomes. Therefore, this study aimed to estimate the prevalence of uncontrolled hypertension and determine the factors associated with poor blood pressure control among patients receiving care at a rural community health centre in the Eastern Cape Province of South Africa.

## 2. Materials and Methods

### 2.1. Study Design

This cross-sectional study was conducted over a three-month period, from August to October 2024, to estimate the prevalence of uncontrolled hypertension and determine the associated factors. Blood pressure control was defined using the universally accepted systolic blood pressure target of <140 mmHg and/or diastolic blood pressure of <90 mmHg [26].

### 2.2. Study Setting

The study was conducted at Mbekweni Community Health Centre, located in a rural area outside Mthatha in the Eastern Cape Province of South Africa. The facility serves patients from Viedgesville, Mqanduli, and surrounding communities. The center operates 24 h a day, although night services are primarily reserved for emergencies.

The Health Care Centre operates 24 h a day; night services are reserved for emergencies. In primary health care facilities in South Africa, all services are offered at no cost to the patients. At initial diagnosis of hypertension, patients are administered an emergency treatment regimen and are requested to visit the facility weekly over the next three weeks. If uncontrolled, the patient is placed on regular treatment and visits the facility monthly for the next three months for ongoing monitoring of blood pressure. After three months of treatment, blood pressure and other laboratory investigations are performed. If still uncontrolled, the patients are scheduled for another review every three months while monthly monitoring continues.

Commonly used antihypertensive medications at the primary care level include diuretics, calcium channel blockers, angiotensin-converting enzyme inhibitors, angiotensin receptor blockers, alpha-blockers, beta-blockers, and vasodilators. The National Department of Health recommends fixed-dose combination therapy, where available, to improve adherence [27].

In South African primary health care facilities, services are provided free of charge to patients. At the initial diagnosis of hypertension, patients commenced on emergency treatment and reviewed weekly for the following three weeks. If blood pressure remains uncontrolled, patients are initiated on regular treatment and reviewed monthly for three months. Thereafter, blood pressure and relevant laboratory investigations are reassessed.

Patients with persistently uncontrolled hypertension are scheduled for review every three months while continuing monthly monitoring. Patients with controlled blood pressure are referred to a Central Chronic Medicines Dispensing and Distribution (CCMDD) center and reviewed every six months.

### 2.3. Population and Sampling Strategy

The study population consisted of a simple random sample of patients receiving clinical management for hypertension at Mbekweni Community Health Centre. Random numbers were generated with the aid of Epidat version 3.1 software on every data collection day. Patients with a diagnosis of hypertension, 18 years or older, who have been reviewed at least twice (first and second review at three months and six months after regular treatment initiation), and those who demonstrated willingness to be part of this study were included. However, patients below 18 years, newly diagnosed, have not been reviewed at least twice, those who were critically ill, those with mental and/or neuropsychiatric disorders, and pregnant women were excluded from this study.

The sample size was determined using the formula:

$$n=\frac{Z_{\alpha }^{2}\cdot P(100-P)}{e^{2}},$$

where *n* represents the required sample size, *Z_α_* is the standard normal deviate corresponding to the chosen confidence level, *P* denotes the estimated prevalence expressed as a percentage, and *e* is the allowable margin of error. Based on a reported prevalence of uncontrolled hypertension of 57% among hypertensive primary healthcare users in Johannesburg [28], a 95% confidence level (Z = 1.96), and a maximum allowable error of 10%, the minimum required sample size was calculated to be 95 participants. To account for potential non-response, an additional 8% was added, resulting in a final target sample size of 103 participants.

### 2.4. Data Collection

A semi-structured questionnaire was administered to obtain sociodemographic (age, gender, marital status), socioeconomic (educational level, employment status, and average monthly income), and lifestyle information (physical activity, smoking status, alcohol consumption, and dietary habits) and information related to health care access and hypertension management. Body mass index (BMI) and clinical variables, including family history of hypertension, duration since diagnosis, systolic blood pressure, and diastolic blood pressure, were obtained from participants’ medical records. Information on medication adherence was collected using the Hill–Bone Compliance to High Blood Pressure Therapy Scale (CHBPTS), a validated tool designed to assess adherence-related behaviours among hypertensive patients. Data collection commenced after university ethical approval and submission of the required documentation to the Department of Health. Approval to proceed while awaiting the official letter was granted due to time constraints for research submission.

### 2.5. Criteria for the Diagnosis of Uncontrolled Hypertension

Uncontrolled hypertension was evaluated based on blood pressure readings on the day of data collection; systolic blood pressure was ≥140 mmHg, and/or diastolic blood pressure was ≥90 mmHg. Hypertension was assessed by measuring blood pressure using a validated automated electronic upper-arm blood pressure monitor (Welch Allyn, Skaneateles Falls, New York, NY, USA) under standardized clinical conditions, in accordance with national and World Health Organization recommendations [27]. Measurements were conducted after participants had rested for at least five minutes in a quiet setting by the third-year medical students under supervision. Participants were seated with back support, feet flat on the floor, legs uncrossed, and arm supported at heart level, using an appropriately sized cuff. Two to three readings of systolic blood pressure (SBP) and diastolic blood pressure (DBP), expressed in millimeters of mercury (mmHg), were taken at 1–2 min intervals, and the average of the final readings was used for analysis.

### 2.6. Inclusion and Exclusion Criteria

Participants were included in the study if they met the following criteria: Adults aged 18 years and older, patients with a confirmed diagnosis of hypertension, receiving follow-up care for hypertension at Mbekweni Community Health Centre, who had been reviewed at least twice after initiation of regular hypertension treatment (specifically at the three-month and six-month follow-up visits), who are willing to participate in the study and provided informed consent.

Participants were excluded in the study if they met the following criteria: Patients younger than 18 years of age, newly diagnosed hypertensive patients who had not yet undergone follow-up reviews, who had not completed at least two follow-up visits after starting regular hypertension treatment, who were critically ill at the time of data collection, pa with mental or neuropsychiatric disorders that could interfere with participation in the study, pregnant women, due to the presence of pregnancy-related hypertension conditions that differ from chronic hypertension.

### 2.7. Definition of Treatment Adherence

The Hill–Bone CHBPTS was developed by the Johns Hopkins University School of Nursing in 1999 and reported Cronbach’s alpha of 0.74, indicating its reliability. The permission to use this scale was obtained. The questionnaire consists of 14 questions across three domains: (1) reducing sodium intake, (2) keeping appointments, and (3) taking medication. Each question is rated on a 4-point Likert scale, where 1 = All the time, 2 = Most of the time, 3 = Some of the time, and 4 = Never. Item 6 was reverse coded so that all items were oriented in the same direction with higher scores consistently indicating better medication adherence. The total score ranges from 14 to 56 was calculated by summing item responses from which means were derived.

Medication adherence was categorized using the sample mean score (≥50 points) on the Hill–Bone Compliance Scale, in the absence of universally established clinical cut-off values, as highlighted in a previous systematic review [29]. Similar approaches have been reported in empirical studies that relied on the distribution of Hill–Bone scores, including percentage-based thresholds (≥80%) [30], or sensitivity-based cut-off values derived using receiver operating characteristic analysis [31]. For this study, good adherence is defined as a score equal to or above the group means Hill–Bone CHBPTS score, while poor adherence is defined as a score below the group mean score.

All information collected through the questionnaire was recorded in Microsoft Excel and then exported to SPSS version 30 (IBM SPSS, Armonk, NY, USA, 2024) for analysis [32]. Qualitative variables were summarized using frequencies and percentages. In contrast, quantitative variables were summarized using the mean and standard deviation if they follow a normal distribution or the median and interquartile range (IQR) if they do not. Tests of association between variables of interest and outcome variable (uncontrolled hypertension) were performed using the Chi-square test. All variables with a significance level of less than 0.05 were entered into the logistic regression model to identify factors associated with uncontrolled blood pressure. A *p*-value < 0.05 was considered statistically significant.

### 2.8. Ethical Considerations

Ethical clearance was obtained from the Research Ethics and Bio-Safety Committee of the Faculty of Medicine and Health Sciences, Walter Sisulu University, with protocol number 123/2024, date 27 August 2024. Data collection commenced after university ethical approval and submission of the required documentation to the Department of Health. Approval to proceed while awaiting the official letter was granted due to time constraints for research submission. Additionally, approval was obtained from the Eastern Cape Provincial Department of Health. Verbal permission was obtained from the operational manager of Mbekweni Community Health Centre. Data collection commenced after university ethical approval and submission of the required documentation to the Department of Health. Approval to proceed while awaiting the official letter was granted due to time constraints for research submission. Written informed consent was obtained from all participants in the study. Research data has no personal identifiers and was stored electronically in a secure file until it was used for analysis.

## 3. Results

One hundred three (*n* = 103) participants were recruited into this study, with a mean age of 63 (standard deviation: 13 years). The majority were females (65%), had attended school up to secondary school (46.6%), were married (58.3%), unemployed (89.3%), and fell within the low household income level of less than R6000 (83.5%). In terms of lifestyle, obesity (70.9%), special diet (61.2%), physical exercise (75.7%), non-smoking (92.2%), non-alcohol drinkers (85.4%), rare use of high sodium foods (48.5%), and less than two servings of fruit per day (59.2%) were predominant among study respondents. Forty (40, 38.8%) had hypertension and diabetes, while 51 (49.5%) had a family history of hypertension (Table 1).

### 3.1. Hypertension Treatment-Related Attributes of the Sampled Participants

Slightly more than half (51.5%) of the participants were diagnosed with hypertension for more than 10 years. All the participants were on medication for hypertension, and the majority (73.8%) always take medications, regularly visit the clinic (96.1%), and had high (52.4%) drug compliance (Table 2). The median drug compliance score was 50 (Interquartile range: 47–52), with the lowest score of 41 and the highest 56. Medication-taking adherence demonstrated the highest median subscale score, followed by sodium-reduction behaviors, while appointment keeping showed comparatively lower adherence. The distribution of responses for the Hill–Bone Compliance Scale items and the correlations between individual items and the three adherence subscales are presented in Supplementary Tables S1 and S2, respectively.

### 3.2. Prevalence of Uncontrolled Hypertensions

**Figure 1:**
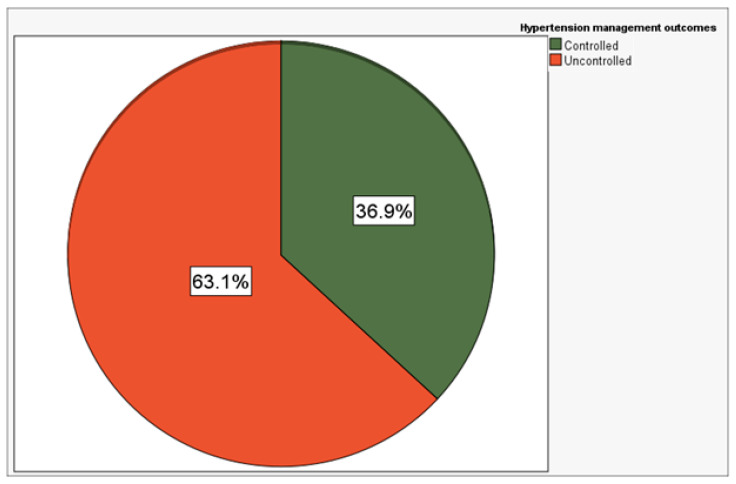
Prevalence of uncontrolled hypertension among 103 patients managed at a community health centre.

Overall, successful treatment occurred in 38 (36.9%) participants while it remained uncontrolled in 65 (63.1%) of the total participants (Figure 1).

**Figure 2:**
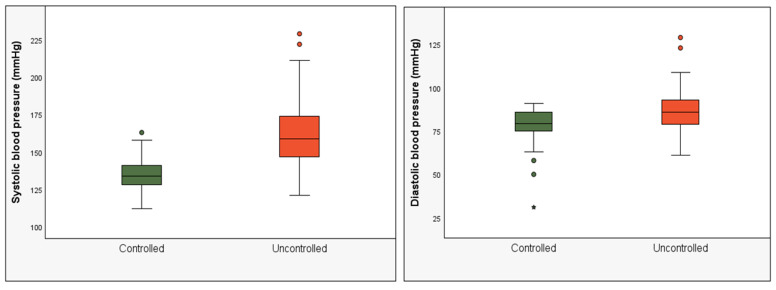
Systolic and diastolic blood pressure readings stratified by hypertension management outcomes. [Boxes (interquartile range with the median line inside); whiskers (values within 1.5 × IQR); ° (outliers); * (extreme outliers); colors (study groups)].

The median systolic blood pressure was 149 mmHg (IQR: 135–163 mmHg), and the median diastolic blood pressure was 84 mmHg (IQR: 76–89 mmHg). The current systolic [159 (147–174) vs. 134 (128–141) mmHg, *p* < 0.001] and diastolic [86 (79–93) vs. 80 (75–86) mmHg, *p* = 0.001] blood pressure reading was significantly higher among participants with uncontrolled hypertension compared to those with controlled hypertension (Figure 2).

### 3.3. Factors Associated with Uncontrolled Hypertension

In the bivariate analysis, only medication adherence was significantly associated with uncontrolled hypertension. No statistically significant difference was observed between hypertension treatment outcomes and sociodemographic, socioeconomic, lifestyle, and clinical parameters (Table 3).

A final multivariable logistic regression was not performed because low medication adherence was the only variable significantly associated with uncontrolled blood pressure in unadjusted analysis. An exploratory analysis including clinically relevant variables and those with *p* < 0.40 was conducted; however, no additional variables remained significant after adjustment (Supplementary Table S3).

**Figure 3:**
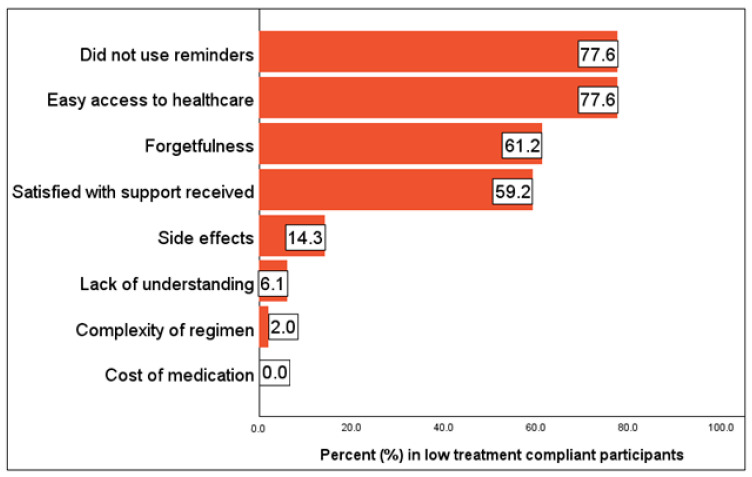
Reported barriers and facilitators to medication adherence among low-compliant participants.

**Figure 4:**
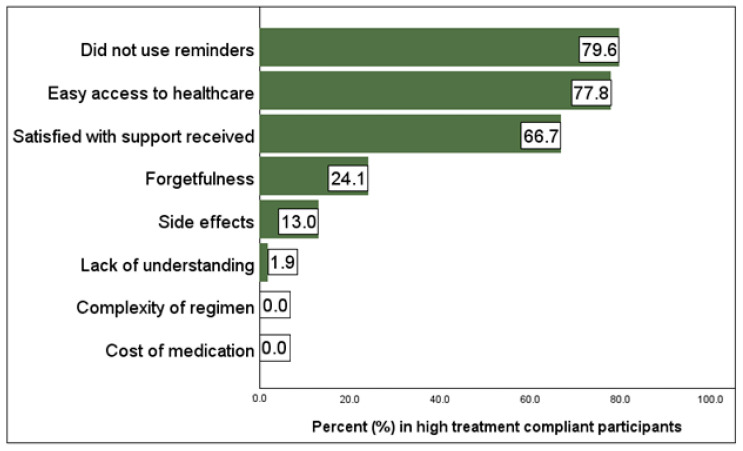
Reported barriers and facilitators to medication adherence among high-compliant participants.

Further analysis showed that among participants with low compliance to hypertension treatment (*n* = 49), a high proportion were not using reminders (77.6%) and often forgot (61.2%) to take medications (Figure 3). No participants with high treatment compliance reported medication complexity (Figure 4).

## 4. Discussion

In South Africa, 8.22 million adults above the age of 18 years were afflicted by hypertension in 2022 [17]. A report by Statistics South Africa in 2023 indicated that half of non-communicable disease-related deaths were due to cardiovascular diseases, with a 46.5% increase from 1997 to 2018, and 90% of these deaths were due to hypertension and 10% to ischaemic heart disease, cerebrovascular disease, and other forms of heart diseases [33]. The demographic profile showed that most hypertensive patients were older adults and female, an attribute that is recurrent in available literature [34,35,36], even though otherwise in other settings [37,38]. The high occurrence with advanced age is well explained using biological processes like stiffening of the aorta that comes with aging [39]. The high rates of hypertension among females have been linked with menopause and use of contraceptives [40] which are biological processes and practices inherent with their gender. Even though there was no significant difference in the ages between males and females in the present study, the fact that most of them were above 60 years could explain why more females than males had hypertension. The median systolic blood pressure was 149 mmHg (interquartile range (IQR) 135–163) while the median diastolic blood pressure was 84 mmHg (IQR 76–89), revealing a predominance of systolic hypertension, a form of hypertension common with advanced age [41,42].

The overall prevalence of uncontrolled hypertension was 63.1%. Compared to other studies conducted outside of Africa, the figure presented in this study was higher than those reported in the United States of America (49.5%) [15] but lower than those reported in Nepal (91.8%). The figure presented in this study was higher than those reported in Zimbabwe (58.6%), but lower than those reported in Nigeria (84.5%) [20], and from a systematic review for sub-Saharan countries (88.5%) [16,43,44,45]. In comparison to other studies conducted in different parts of South Africa, the result is still higher than the 56.83% prevalence that was reported among adult residents of Mkhondo municipality 49.8% in a rural community health centre in Gauteng province and 53.7% reported in a population-based study across South African provinces. It is important to note that the reported rate in the present study reflects a 12.4% decline from 75.5% previously reported in a survey conducted in the region in 2016 [39,46,47]. The observed decline may be partly associated with the relatively high proportion of participants reporting regular clinical visits and healthier self-management practices; however, this relationship cannot be interpreted causally. The finding revealed that high proportions were on special diets, rarely used high-sodium food, non-smokers, non-drinkers, and did not live a sedentary lifestyle. Hypertensive patients who engage in unhealthy lifestyle practices have been reported to have uncontrolled hypertension [48,49]. Additionally, having other medical conditions, particularly type 2 diabetes mellitus, was found to increase the chances of uncontrolled hypertension in other populations [50]. Still, very few cases of comorbidity were observed in the present study. The relatively low occurrence of these factors may have been observed alongside the lower proportion of uncontrolled hypertension, although no causal relationship can be inferred. Nonetheless, the 36.9% hypertension control rate is below the expected 50% by the national department of health and far below the 80% set in Sustainable Development Goal 3 (SDG 3) target four. SDG 3 aims to ensure healthy lives and promote well-being for all ages, while target 3.4 focuses on reducing premature mortality from non-communicable diseases by one-third through prevention, treatment, and promotion of mental health and well-being by 2030. We further clarified that improving hypertension diagnosis, treatment adherence, and blood pressure control are essential components of achieving this global health target. In the present study, 52.4% of the participants were fully adherent to hypertensive treatment, which is higher than 42.0% reported in another rural community health centre in South Africa [51]. Although demographic, clinical, and lifestyle factors have been linked to poor control of hypertension these factors did not influence the hypertension control in the present study [16,52,53]. The only significant factor associated with uncontrolled hypertension was low compliance with treatment (OR = 6.02, *p* = 0.001). The association between low compliance and uncontrolled hypertension was also documented in previous international and national literature. The observed blood pressure control rate reflects outcomes within a single rural primary health care facility and should not be interpreted as representative of the wider Eastern Cape or South African hypertensive population. Factors contributing to low compliance with antihypertensive treatment include complex regimens and multiple doses [54]. Specifically, this study assessed adherence using the 14 questions in the validated Hill–Bone compliance scale, which looks at medication side effects and pill burden. The adherence scale is a relatively subjective, non-invasive, and cost-effective tool with limitations [55]. For example, it assesses hypertension treatment behaviour rather than adherence based on actual ingestion of the antihypertensive drug. The preceding finding may indicate why the level of adherence was slightly higher compared to actual blood pressure control rates (52.4% vs. 36.9%). However, as already discussed, the reported blood pressure control rate reflects an improvement from previous rates [56]. Further analysis reveals no significant association of demographic, clinical, and lifestyle factors with medication compliance, unlike drug unavailability and unaffordability contributing to its occurrence in other settings [23,57,58]. In South Africa, primary health care services, including consultation, diagnosis, and treatment administration, are offered at no cost to the patient, making drug affordability an unlikely cause of non-adherence. Additionally, the findings confirm that a minimal proportion of participants had issues with accessing the facility, side effects of medication, complexity of drugs, understanding the treatment regimen, and were satisfied with the support they received from the facility. Forgetfulness and non-use of reminders were commonly reported among participants with low compliance and may represent important correlations of reduced adherence. These could be due to a high proportion of persons 70 and older with uncontrolled hypertension and the observed 4.33-fold risk (95% CI: 0.50–37.26) compared to younger age patients. Forgetfulness in taking medication remains a significant contributor to treatment non-compliance, particularly with advancing age caused by cognitive deficits [59]. The non-use of reminders significantly contributes to low compliance in the present study, highlighting the need to incorporate novel interventions such as digital health tools and treatment buddies to improve adherence behaviour among patients on antihypertensive treatments in rural areas. Digital health interventions, including motivational strategies for feedback, health literacy, reminders, motivational messages, goal setting, social interaction, gamification, and rewards, are suggested measures that could improve adherence to self-care behaviours among patients with chronic diseases [60]. The use of treatment buddies has also contributed to improved adherence in patients with mental health disorders. Considering the age of the participants, coupled with the issue of forgetfulness, assigning treatment buddies in conjunction with digital health tools could be an appropriate intervention.

### 4.1. Strengths and Limitations

This study is significant as it confirms a shift from known sociodemographic, socioeconomic, and lifestyle determinants of uncontrolled hypertension in rural settings. The findings also highlight the main reasons for poor compliance, which warrant insights on improving adherence to antihypertensive treatment through digital health interventions and other measures. The study provides context-specific evidence that may assist healthcare professionals and local health planners in identifying gaps in hypertension management within comparable rural primary health care settings. Despite the revelations of valuable insights that could improve hypertension treatment outcomes, this study has several limitations that should be acknowledged. First, the research was confined to participants from a community health centre that provides health services to a predominantly rural population from Mbekweni. This delimitation may limit the generalizability of the findings to other parts of the Eastern Cape or South Africa, particularly peri-urban and urban areas. Additionally, the study relied on self-reported data, which can be subject to recall bias and social desirability bias, particularly when assessing lifestyle practices and medication adherence. Moreover, since the study used a cross-sectional design, interpretation regarding the causal relationship between uncontrolled hypertension and the mentioned associated factors is limited. Future research endeavours should address these limitations to improve findings on the prevalence and related factors of uncontrolled hypertension. The participants were recruited from a single rural community health center; therefore, comparisons with national or global hypertension control benchmarks should be interpreted cautiously and primarily as contextual reference points rather than direct indicators of broader health system performance. The sample size was sufficient for prevalence estimation and exploratory analyses but may have limited power to detect smaller associations. The use of a sample-derived cut-off to categorize Hill–Bone medication adherence represents a methodological limitation and may reduce comparability with findings from other settings.

### 4.2. Recommendations

The observed blood pressure control rate of 36.9% in this rural primary health care facility remains below levels considered desirable in national and global hypertension control initiatives, which emphasize substantial improvements in diagnosis, treatment adherence, and long-term control of hypertension within primary care settings. While the present findings are limited to a single clinic’s population and are not nationally representative, they highlight the need for strengthened adherence support strategies in similar underserved settings.

## 5. Conclusions

This study provides context-specific evidence on the high prevalence of uncontrolled hypertension among treated adults attending a rural primary health care facility in the Eastern Cape Province of South Africa. The findings contribute to the limited body of evidence on hypertension control in underserved rural communities, where socioeconomic disadvantage and health system constraints may negatively influence long-term disease management. A key novel contribution of this study is the identification of medication adherence, particularly forgetfulness and non-use of reminders, as the main factor associated with uncontrolled hypertension within this setting, while commonly reported sociodemographic and lifestyle factors were not significantly associated with blood pressure control. The study highlights the ongoing challenge of achieving adequate hypertension control in resource-constrained primary health care environments despite regular clinic attendance and availability of free health services. These findings underscore the importance of strengthening adherence-support interventions tailored to older rural populations. The results further suggest that low-cost and scalable strategies such as digital reminder systems, treatment buddy programmes, patient education, and community-based adherence support may play an important role in improving long-term blood pressure control. Future research should focus on longitudinal and multi-centre studies involving larger and more diverse populations to better understand causal pathways influencing hypertension control in rural South African communities. Although these findings arise from a single rural clinic and should not be generalized to all South African hypertensive populations, the relatively low blood pressure control rate underscores persistent challenges in achieving optimal hypertension management in resource-constrained primary care environments. Low compliance was found to be the only associated factor with uncontrolled hypertension. Forgetfulness and non-use of reminders were the most reported factors observed among participants with low compliance.

## Figures and Tables

**Table 1:** Sociodemographic, socioeconomic, and lifestyle characteristics stratified by level of hypertension control.

Variables	Categories	*n* (%) *N* = 103
Sociodemographic		
Age group	30–39	4 (3.9)
	40–49	15 (14.6)
	50–59	21 (20.4)
	60–69	31 (30.1)
	≥70	32 (31.1)
Age	Years	63 ± 13
Gender	Male	36 (35.0)
	Female	67 (65.0)
Educational level	No formal education	14 (13.6)
	Primary education	37 (35.9)
	Secondary education	48 (46.6)
	Higher education	4 (3.9)
Marital status	Single	23 (22.3)
	Married	60 (58.3)
	Divorced	0 (0.0)
	Widowed	20 (19.4)
Socioeconomic		
Employment status	Employed	11 (10.7)
	Unemployed	92 (89.3)
Household income level (per month)	Low [<R6000]	86 (83.5)
	Middle [R6001–R15,000]	15 (14.6)
	High [>R15,000]	2 (1.9)
Lifestyle		
Nutritional status	Normal body mass index	30 (29.1)
	Obese	73 (70.9)
Special diet	No	40 (38.8)
	Yes	63 (61.2)
Exercise	No	25 (24.3)
	Yes	78 (75.7)
Smoking	No	95 (92.2)
	Yes	8 (7.8)
Alcohol consumption	No	88 (85.4)
	Yes	15 (14.6)
Frequency of high-sodium foods	Daily	15 (14.6)
	Weekly	13 (12.6)
	Monthly	15 (14.6)
	Rarely	50 (48.5)
	Never	10 (9.7)
Servings of fruit per day	0–1	61 (59.2)
	2–3	42 (40.8)
Clinical		
Diabetes	No	63 (61.2)
	Yes	40 (38.8)
Family history of hypertension	No	52 (50.5)
	Yes	51 (49.5)

**Table 2:** Hypertension treatment-related attributes.

Variables of Interest	Categories	*n* (%) *N* = 103
Time of diagnosis with hypertension	Less than 1 year	1 (1.0)
	1–5 years	27 (26.2)
	6–10 years	22 (21.4)
	More than 10 years	53 (51.5)
On medication for hypertension	No	0 (0.0)
	Yes	103 (100.0)
Frequency of taking medication	Always	76 (73.8)
	Often	21 (20.4)
	Sometimes	4 (3.9)
	Rarely	1 (1.0)
	Never	1 (1.0)
Regular clinic visit	No	4 (3.9)
	Yes	99 (96.1)
Medication adherence	Low compliance	49 (47.6)
	High compliance	54 (52.4)
Medication adherence score	Median (Interquartile range)	50 (47–52)
Subscale: Reducing sodium intake	Median (IQR)	10 (9–11)
Subscale: Appointment keeping	Median (IQR)	5 (5–7)
Subscale: Medication taking	Median (IQR)	35 (33–36)

**Table 3:** Bivariate associated factors of uncontrolled hypertension.

Variables	Categories	Unadjusted Odds Ratio [95% CI]	Significant Level
Age group	30–39	Ref	0.164
	40–49	1.50 [0.16–13.75]	0.720
	50–59	1.33 [0.16–11.36]	0.792
	60–69	1.07 [0.13–8.56]	0.952
	≥70	4.33 [0.50–37.26]	0.182
Gender	Male	Ref	
	Female	1.64 [0.71–3.76]	0.246
Educational level	No formal education	Ref	0.403
	Primary education	0.57 [0.13–2.42]	0.445
	Secondary education	0.35 [0.09–1.42]	0.142
	Higher education	0.27 [0.03–2.83]	0.276
Employment status	Employed	Ref	
	Unemployed	2.25 [0.64–7.95]	0.208
Marital status	Single	Ref	0.497
	Married	0.61 [0.22–1.71]	0.349
	Widowed	1.02 [0.28–3.77]	0.975
Household income per month	Low [<R6000]	Ref	0.638
	Middle [R6001–R15,000]	0.61 [0.20–1.85]	0.385
	High [>R15,000]	0.54 [0.03–8.87]	0.663
Nutritional status	Normal weight	Ref	
	Obese	1.47 [0.62–3.50]	0.386
Special diet	No	Ref	
	Yes	0.51 [0.22–1.19]	0.118
Exercise	No	Ref	
	Yes	0.59 [0.22–1.58]	0.293
Smoking	No	Ref	
	Yes	1.83 [0.35–9.56]	0.473
Alcohol consumption	No	Ref	
	Yes	1.20 [0.38–3.82]	0.757
Frequency of high-sodium foods	Daily	Ref	0.622
	Weekly	0.00 [0.00--]	1.000
	Monthly	0.00 [0.00--]	0.999
	Rarely	0.00 [0.00--]	1.000
Servings of fruit per day	0–1 servings	Ref	
	2–3 servings	1.09 [0.48–2.47]	0.837
Time of diagnosis with hypertension	Less than 1 year	0.00 [0.00–0.00]	0.475
	1–5 years	0.70 [0.27–1.80]	0.456
	6–10 years	1.49 [0.50–4.46]	1.000
	More than 10 years	Ref	0.674
Frequency of taking medication	Always	0.00 [0.00--]	0.377
	Often	0.00 [0.00--]	1.000
	Sometimes	0.00 [0.00--]	1.000
	Rarely	0.00 [0.00--]	1.000
	Never	Ref	1.000
Diabetes	No	Ref	
	Yes	1.14 [0.50–2.61]	0.751
Family history of hypertension	No	Ref	
	Yes	0.69 [0.31–1.55]	0.373
Regular clinic visit	No	Ref	
	Yes	1.75 [0.24–12.96]	0.584
Medication adherence	Low compliance	4.20 [1.75–10.09]	0.001
	High compliance	Ref	
Hill–Bone total score	Controlled|Uncontrolled	51 (49–53)|49 (47–51)	0.004
Reducing sodium intake	Controlled|Uncontrolled	10.5 (10–11)|10 (9–11)	0.036
Appointment keeping	Controlled|Uncontrolled	5 (5–5)|5 (5–7)	0.927
Medication taking	Controlled|Uncontrolled	36 (34–36)|34 (32–35)	0.003

## Data Availability

The data presented in this study are available upon reasonable request from the corresponding author.

## Supplementary Materials

The following supporting information can be downloaded at https://www.mdpi.com/article/10.3390/epidemiologia7030082/s1, Table S1: Distribution of Responses for Hill Bone Compliance Scale Items; Table S2: Spearman’s rank correlations between individual Hill Bone items and the three adherence subscales; Table S3: Multivariable Logistic Regression Analysis of Factors Associated with Uncontrolled Hypertension.

## Abbreviations

The following abbreviations are used in this manuscript:

BMI	Body Mass Index
CCMDD	Central Chronic Medicines Dispensing and Distribution
CHBPTS	Hill–Bone Compliance to High Blood Pressure Therapy Scale
mmHg	Millimetres of Mercury
NCD	Non-Communicable Disease
PHC	Primary Health Care
WHO	World Health Organization
SPSS	Statistical Package for the Social Sciences
