# Supplementary material for: Prevalence and Determinants of Uncontrolled Hypertension Among Treated Adults in a Rural Primary Health Care Facility in South Africa: A Cross-Sectional Study
Source: Epidemiologia (Basel). 2026 Jun 10;7(3):82. doi: 10.3390/epidemiologia7030082 (PMC13298852; doi:10.3390/epidemiologia7030082)
Supplement: Supplementary file 1 [file epidemiologia-07-00082-s001.zip › epidemiologia-4213489-supplementary.pdf]

Supplementary Table S1. Distribution of Responses for Hill-Bone Compliance Scale Items

Hill-Bone Compliance Items	All the Time n (%)	Most of the Time n (%)	Sometimes n (%)	Never n (%)	Median (IQR)
1.How often do you forget to take your high blood pressure medicine?	0 (0.0)	3 (2.9)	40 (38.8)	60 (58.3)	4 (3–4)
2.How often do you decide NOT to take your high blood pressure medicine?	0 (0.0)	0 (0.0)	18 (17.5)	85 (82.5)	4 (4–4)
3.How often do you eat salty food?	4 (3.9)	19 (18.4)	49 (47.6)	31 (30.1)	3 (3–4)
4.How often do you shake salt on your food before you eat it?	2 (1.9)	7 (6.8)	13 (12.6)	81 (78.6)	4 (4–4)
5.How often do you eat fast food?	3 (2.9)	8 (7.8)	57 (55.3)	35 (34.0)	3 (3–4)
6.How often do you make the next appointment before you leave the doctor's office?	70 (68.0)	1 (1.0)	6 (5.8)	26 (25.2)	1 (1–4)
7.How often do you miss scheduled appointments?	5 (4.9)	2 (1.9)	28 (27.2)	68 (66.0)	4 (3–4)
8.How often do you forget to get prescriptions filled?	0 (0.0)	2 (1.9)	6 (5.8)	95 (92.2)	4 (4–4)
9.How often do you run out of high blood pressure pills?	0 (0.0)	1 (1.0)	31 (30.1)	71 (68.9)	4 (3–4)
10.How often do you skip your high blood pressure medicine before you go to the doctor?	4 (3.9)	9 (8.8)	16 (15.7)	73 (71.6)	4 (3–4)
11.How often do you miss taking your high blood pressure pills when you feel better?	0 (0.0)	1 (1.0)	14 (13.6)	88 (85.4)	4 (4–4)
12.How often do you miss taking your high blood pressure pills when you feel sick?	0 (0.0)	0 (0.0)	3 (2.9)	100 (97.1)	4 (4–4)
13.How often do you take someone else's high blood pressure pills?	0 (0.0)	1 (1.0)	4 (3.9)	98 (95.1)	4 (4–4)
14.How often do you miss taking your high blood pressure pills when you are careless?	1 (1.0)	2 (1.9)	24 (23.3)	76 (73.8)	4 (3–4)

Supplementary Table S2. Spearman's rank correlations between individual Hill-Bone items and the three adherence subscales

Items demonstrated stronger correlations with their theoretically assigned subscales than with other domains, supporting the construct validity of the three-domain structure of the Hill-Bone Compliance Scale.

	Subscale groups		
Hill-Bone Compliance Items	Reducing sodium intake (rho)	Appointment keeping (rho)	Medication taking (rho)

How often do you forget to take your high blood pressure medicine?	0.071	-0.035	0.729**
How often do you decide NOT to take your high blood pressure medicine?	-0.036	0.076	0.504**
How often do you eat salty food?	0.841**	0.112	0.117
How often do you shake salt on your food before you eat it?	0.602**	0.202*	0.152
How often do you eat fast food?	0.693**	0.119	0.099
How often do you make the next appointment before you leave the doctor's office?*	0.130	0.810**	-0.064
How often do you miss scheduled appointments?	0.171	0.514**	0.325**
How often do you forget to get prescriptions filled?	0.124	-0.054	0.265**
How often do you run out of high blood pressure pills?	-0.035	0.035	0.425**
How often do you skip your high blood pressure medicine before you go to the doctor?	0.193	-0.031	0.681**
How often do you miss taking your high blood pressure pills when you feel better?	0.059	0.067	0.538**
How often do you miss taking your high blood pressure pills when you feel sick?	0.091	0.037	0.205*
How often do you take someone else's high blood pressure pills?	0.062	0.004	0.285**
How often do you miss taking your high blood pressure pills when you are careless?	0.242*	0.249*	0.569**

rho= Spearman's rank correlation coefficient. * denotes statistical significance at the 0.05 level** denotes statistical significance at the 0.01 level

Supplementary Table S3. Multivariable Logistic Regression Analysis of Factors Associated with Uncontrolled Hypertension

Variables of interest	Adjusted odds ratio (95%CI)	Significant level
Age Group: ≥60 years vs 60 years	1.12 (0.41–3.05)	0.831
Gender: Female vs Male	1.69 (0.66–4.33)	0.276
Employment status: Unemployed vs Employed	2.34 (0.50–10.97)	0.281
Nutritional status: Obese vs Normal weight	2.24 (0.78–6.40)	0.132
Special diet: No vs Yes	1.33 (0.46–3.85)	0.595
Exercise: No vs Yes	2.22 (0.71–6.96)	0.172
Family history of hypertension: No vs Yes	2.03 (0.79–5.24)	0.143
Medication adherence: Low compliance vs High compliance	5.25 (1.89–14.57)	0.001
